# Lin28a promotes self-renewal and proliferation of dairy goat spermatogonial stem cells (SSCs) through regulation of mTOR and PI3K/AKT

**DOI:** 10.1038/srep38805

**Published:** 2016-12-12

**Authors:** Fanglin Ma, Zhe Zhou, Na Li, Liming Zheng, Chongyang Wu, Bowen Niu, Furong Tang, Xin He, Guangpeng Li, Jinlian Hua

**Affiliations:** 1 College of Veterinary Medicine, Shaanxi Centre of Stem Cells Engineering & Technology, Northwest A&F University, Yangling, Shaanxi, 712100 China; 2 Key Laboratory for Mammalian Reproductive Biology and Biotechnology, Ministry of Education, Inner Mongolia University, Hohhot, 010021, China

## Abstract

Lin28a is a conserved RNA-binding protein that plays an important role in development, pluripotency, stemness maintenance, proliferation and self-renewal. Early studies showed that Lin28a serves as a marker of spermatogonial stem cells (SSCs) and promotes the proliferation capacity of mouse SSCs. However, there is little information about Lin28a in livestock SSCs. In this study, we cloned *Capra hircus* Lin28a CDS and found that it is evolutionarily conserved. Lin28a is widely expressed in different tissues of Capra hircus, but is expressed at a high level in the testis. Lin28a is specifically located in the cytoplasm of *Capra hircus* spermatogonial stem cells and may also be a marker of dairy goat spermatogonial stem cells. Lin28a promoted proliferation and maintained the self-renewal of GmGSCs-I-SB *in vivo* and *in vitro*. Lin28a-overexpressing GmGSCs-I-SB showed an enhanced proliferation rate, which might be due to increased PCNA expression. Moreover, Lin28a maintained the self-renewal of GmGSCs-I-SB by up-regulating the expression of OCT4, SOX2, GFRA1, PLZF and ETV5. Furthermore, we found that Lin28a may activate the AKT, ERK, and mTOR signaling pathways to promote the proliferation and maintain the self-renewal of GmGSCs-I-SB.

Lin28, a conserved RNA-binding protein, was first characterized in *Caenorhabditis elegans* as a heterochronic gene that controls developmental timing^1^,^2^. Lin28 is highly expressed in embryonic stem cells (ESCs), but its expression is directly repressed by Lin-4 and Let-7 miRNA upon differentiation^2^,^3^. Lin28 contains a cold shock domain (CSD) and two zinc-binding motifs (CCHCx2), which are two important RNA interaction domains 2. There are two homologs in mammals named Lin28a and Lin28b that are located both in the cytoplasm and nucleus^4^, and these proteins have parallel functions in many respects.

Lin28 expression was directly repressed in ESCs by Lin-4 and Let-7 miRNA upon differentiation^2^,^3^. However, Let-7 microRNA biogenesis was also repressed by Lin28 4. The Lin28/Let-7 feedback loop plays an important role in many physiological activities. Lin28a binds to and suppresses mRNA translation in ESCs^5^. In addition, Lin28a also binds to and enhances the translation of some mRNAs, such as OCT4 in ESCs, IGF2 in myoblasts and several metabolic enzymes^6^,^7^,^8^,^9^,^10^. Glucose tolerance and insulin resistance were impaired in Lin28a knockout mice, and the expression of several transcription factors was altered by Lin28a-overexpression in early embryonic cells^11^,^12^.

SSCs are undifferentiated male germ cells that transmit genetic material to the next generation^13^,^14^. SSCs exist in seminiferous tubules, where SSCs perform spermatogenesis throughout adult life to maintain male fertility. SSCs in adult male testis balance self-renewal and differentiation to maintain spermatogenesis^13^,^15^. However, to date, little is known about the identity of SSCs due to the lack of adequate specific markers. In addition, the identity and function of livestock SSCs are even more unclear.

Recently, studies have demonstrated that Lin28a might be a marker of spermatogonial progenitor populations. In adult mouse testes, Lin28a was expressed in undifferentiated spermatogonia^16^. Lin28a-positive germ cells are spermatogonial stem cells in hamster and monkey. Moreover, Lin28a act as an intrinsic regulator of proliferation of spermatogonia^17^. Clonal expansion of progenitor TA, A undifferentiated spermatogonia was impaired when Lin28a was conditionally deleted in the adult male mouse germline^17^. However, the signaling pathway involved in Lin28a function in spermatogonia is still unknown.

Mammalian target of rapamycin (mTOR) is a serine-threonine protein kinase that belongs to the phosphatidylinositol kinase-related kinase family^18^,^19^. AKT and mTOR are the key signals that regulate the balance between self-renewal and differentiation of SSCs^20^. The Ras/ERK1/2 signaling pathway is an important pathway that has a vital role in cell proliferation, differentiation, and cell cycle progression^21^,^22^. The activated Ras/ERK1/2 pathway promotes the maintenance and self-renewal of dairy goat SSCs^23^.

Dairy goat is the subspecies of *Capra hircus* that is important in Chinese life. Dairy goat has an important economic value as it can provide abundant meat, wool and dairy. Therefore, improving preservation and optimizing germplasm resources are important. In this study, the expression pattern of Lin28a and its function in Guanzhong dairy goat SSCs were investigated. The expression of OCT4, SOX2, GFRA1, PLZF, ETV5 and PCNA in the GmGSCs-I-SB was up-regulated in dairy goat male germline stem cells when Lin28a was overexpressed. Thus, Lin28a is potentially essential for the self-renewal and proliferation of dairy goat male germline stem cells. Moreover, AKT, ERK, mTOR and S6 were also activated in dairy goat male germline stem cells that overexpressed Lin28a. Thus, we hypothesized that Lin28a may maintain self-renewal and promote proliferation of dairy goat mGSCs through the regulation of PI3K/AKT, ERK and mTOR.

## Results

### Lin28a expression in dairy goat

**Figure 1:**
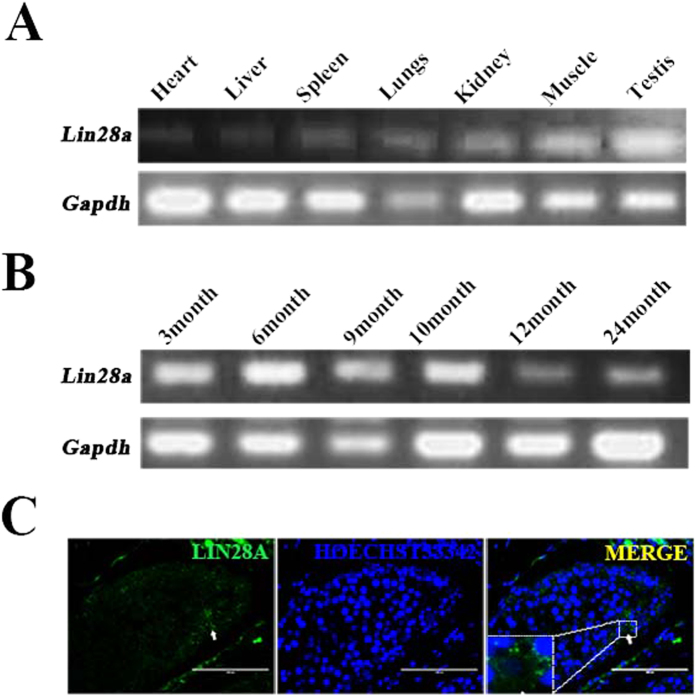
Expression pattern of Lin28a in *Capra hircus*. (**A**) Expression of Lin28a in different tissues of *Capra hircus* as measured by semi-quantitative PCR. (**B**) Semi-quantitative PCR analysis of the expression of Lin28a in dairy goat testes at different ages. (**C**) The location of Lin28a in dairy goat testis analyzed by immunofluorescence. Lin28a located in the cytoplasm of dairy goat spermatogonial stem cells (gSSCs). Bar = 100 μm.

Semi-quantitative RT-PCR analysis showed that Lin28a is widely expressed in various dairy goat organs, including the testis, lung, heart, liver, spleen, kidney and muscle. Among these tissues, Lin28a expression levels in testis were high (Fig. 1A), and Lin28a expression was high in pubertal testes (Fig. 1B). Immunofluorescence staining showed that Lin28a is located in the cytoplasm of the dairy goat spermatogonia and spermatogonial stem cells (gSSCs) (Fig. 1C).

### Cloning and bioinformatics analysis of Lin28a

**Figure 2:**
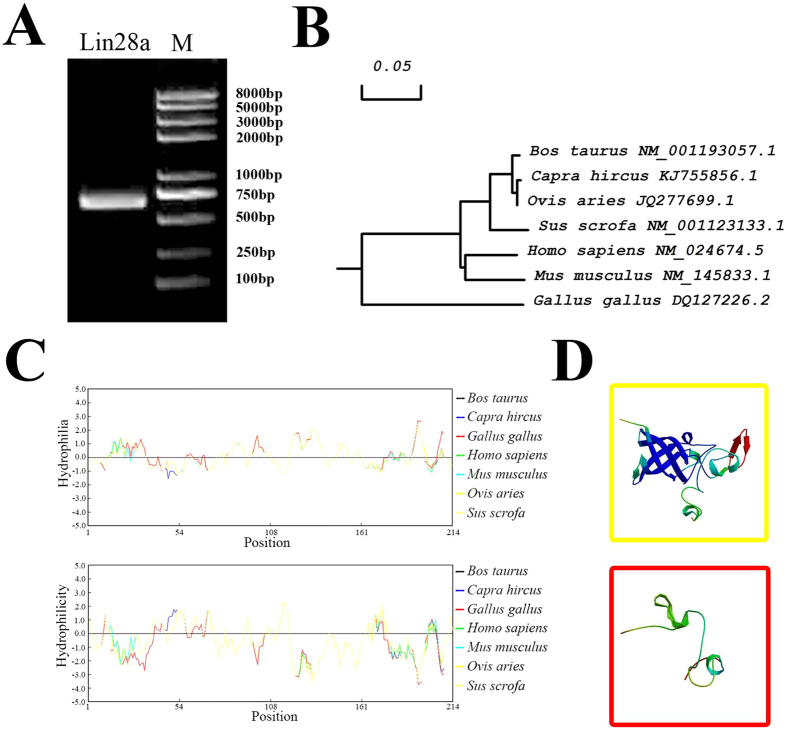
Identification of *Capra hircus* Lin28a gene. (**A**) Lin28a cloned. (**B**) Phylogenetic tree of Lin28a constructed by MEGA4. (**C**) Hydrophilicity (up) and hydrophobicity (down) analyses of *Capra hircus* Lin28a by DNAMAN. (**D**) Prediction of domains in *Capra hircus* Lin28a: an S1-like cold-shock domain (CSD) (up) and two the Zinc-finger domains (down).

The Lin28a gene was cloned from dairy goat testis cDNA by PCR. Fragments from 500 bp to 750 bp, which we assumed to be the Lin28a gene, were obtained (Fig. 2A). Then, we cloned the fragments into the pMD18-T vector for sequencing. The sequencing results indicated that the CDS of *Capra* goat Lin28a gene was 630 bp (Figure S1). Furthermore, we submitted the sequence to National Center for Biotechnology Information (NCBI) and obtained the formal sequence number (KJ755856).

We aligned the nucleic acid sequences and amino acid sequences for *Bos taurus*, *Ovis aries* and other species from NCBI by DNAMAN. The results were also verified by a phylogenetic tree constructed by MEGA4 24 (Fig. 2B), indicating that Lin28a is conserved in these species (Table S1, Figure S2A). Hydrophobicity and hydrophilicity play important roles in protein structure. These features were all located in the middle of a highly conserved area among the different species (Fig. 2C). In fact, the structure contains the important RNA binding domains of Lin28a: a cold shock domain (CSD) and two tandem zinc-binding motifs (CCHCx2). These findings indicate that these domains may be present in the *Capra hircus* Lin28a protein. A SWISS-MODEL Workspace prediction also showed that the two RNA binding motifs were present (Fig. 2D, Figure S2B,C). These results indicate that the Lin28a protein is conserved and might have similar functions in different cells and species.

### Lin28a overexpression in GmGSCs-I-SB cells

**Figure 3:**
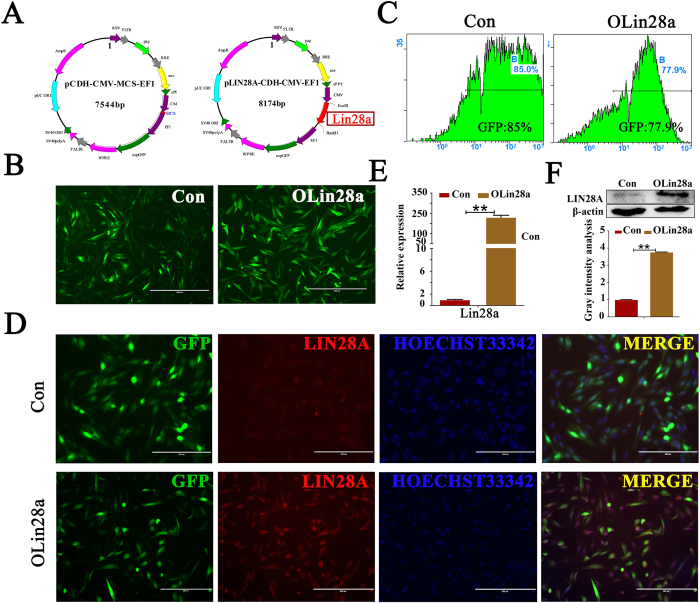
Establishment of GmGSCs-I-SB-pCDH and GmGSC-I-SB-Lin28a cell line. (**A**) Lentivirus plasmid vector pCDH-CMV-MSC-EF1 and recombinant plasmid pLIN28A-CDH-CMV-MSC-EF1; (**B**) construction GmGSCs-I-SB-GFP and GmGSCs-I-SB-Lin28a cell; Bar = 200 μm; (**C**). GFP fluorescence intensity analysis of the two cell lines, as measured by FCM (flow cytometry). (**D**) Immunofluorescent staining detected the expression of Lin28a in the two cells. Bar = 200 μm. (**E**) RT-PCR analysis of the expression of Lin28a in GmGSCs-I-SB-GFP and GmGSCs-I-SB-Lin28a cells. Lin28a was overexpressed in GmGSCs-I-SB-Lin28a cells at the mRNA level. (**F**) Western blot analysis of Lin28a expression levels in GmGSCs-I-SB-GFP and GmGSCs-I-SB-Lin28a cells. Lin28a was overexpressed in GmGSCs-I-SB-Lin28a cells at the protein level. **p < 0.01.

The Lin28a fragment was inserted into the pCDH-CMV-MSC-EF1 plasmid, and the pLin28-CDH-CMV-EF1 recombination plasmid was constructed (Fig. 3A). The recombination plasmid was verified by EcoRI and BamHI double digestion. Then, the two plasmids were transduced into GmGSCs-I-SB cells with PAX2 and VSVG plasmids. The GFP-positive cells were observed 12 h after transduction. Then, the GFP-positive cells were screened and purified by puromycin dihydrochloride to obtain more intensive GFP-labeled cells. The percentage of GFP-positive cells in the OLin28a group was 77.9%, and the percentage in the control group was 85% (Fig. 3B,C). Subsequently, transcription and translation of Lin28a in the two groups were detected. Lin28a was significantly increased in the Lin28a-transduced group compared with the control group (Fig. 3D,E,F). These results indicate that the recombination plasmid and lentivirus system were effective and that Lin28a was overexpressed in the transduced group.

### Lin28a promotes the proliferation and self-renewal of dairy goat mGSCs

**Figure 4:**
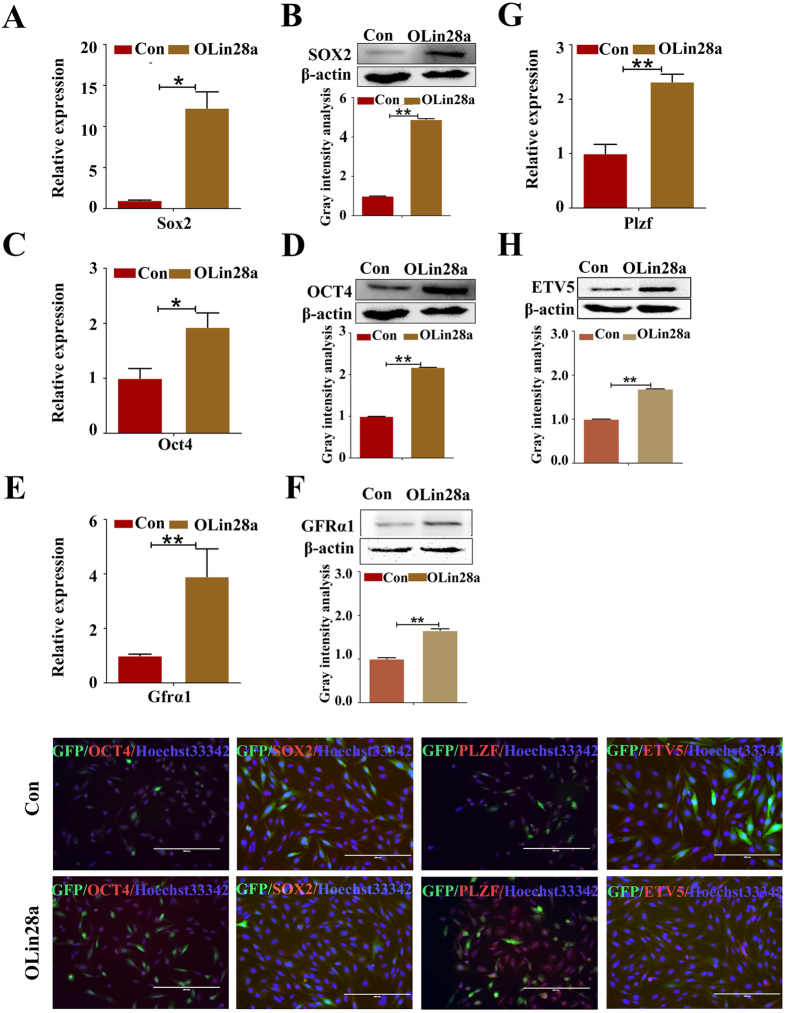
Lin28a enhanced the stemness and self-renewal of GmGSC-SB cells. (**A,C,E,G**) RT-PCR analysis of the expression of SOX2, OCT4, GFRΑ1 and PLZF in GmGSCs-I-SB-GFP and GmGSCs-I-SB-Lin28a cells. SOX2, OCT4, GFRΑ1 and PLZF were highly expressed in GmGSCs-I-SB-Lin28a cells at the mRNA level. (**B,D,F,H**) Western blot analysis of SOX2, OCT4, GFRA1 and ETV5 expression levels in GmGSCs-I-SB-GFP and GmGSCs-I-SB-Lin28a cells. SOX2, OCT4, GFRA1 and ETV5 were highly expressed in GmGSCs-I-SB-Lin28a cells at the protein level. I. Immunofluorescent staining of SOX2, OCT4, PLZF and ETV5. Bar = 200 μm; *p < 0.05,**p < 0.01.

Lin28a has emerged as a novel factor that defines stemness in recent years^24^,^25^. Thus, we assumed that Lin28a could maintain the self-renewal and proliferation of dairy goat SSCs. To confirm our assumption, the expression levels of pluripotency markers (i.e., OCT4 and SOX2) in GmGSCs-I-SB cells and overexpressed Lin28a cells were examined. The results showed that the OCT4 and SOX2 expression levels were significantly increased in the transduced Lin28a group (OLin28a) compared with the control group (Con) (Fig. 4A,B,C,D).

Proliferation and self-renewal are the fundamental characters of SSCs^26^. To investigate the self-renewal of cells in the two groups, the expression levels of the SSC markers GRFA1, PLZF, OCT4 and ETV5 were tested. GRFA1, PLZF, OCT4 and ETV5 expression was increased in the OLin28a group compared with the Con group (Fig. 4E,F,G,H). These results were also verified by the immunofluorescence analysis (Fig. 4I).

**Figure 5:**
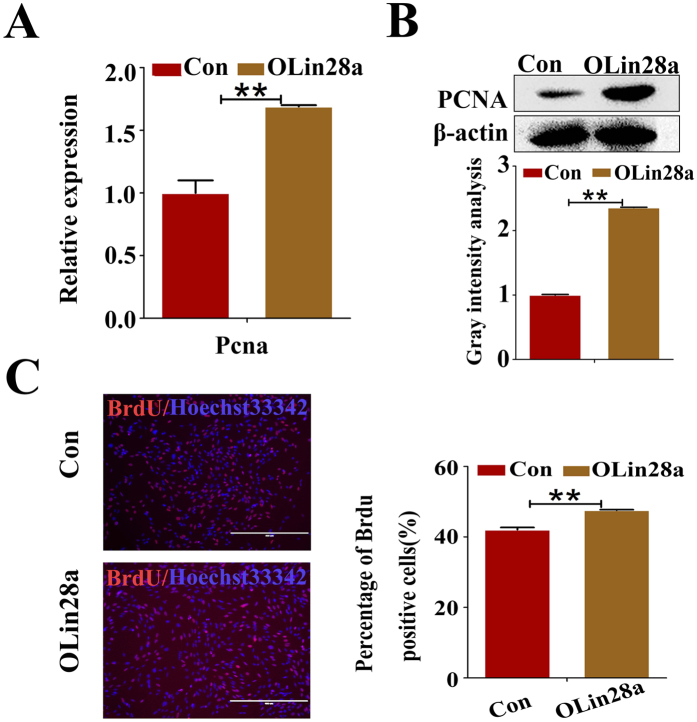
Lin28a promotes GmGSCs-SB cell proliferation. (**A**) RT-PCR analysis of PCNA expression in GmGSCs-I-SB-GFP and GmGSCs-I-SB-Lin28a cells. (**B**) Western blot analysis the expression of PCNA in GmGSCs-I-SB-GFP and GmGSCs-I-SB-Lin28a cells. (**C**) BrdU assay analysis the proliferation level of GmGSCs-I-SB-GFP and GmGSCs-I-SB-Lin28a cells. Bar = 200 μm **p < 0.01.

The expression level of proliferating cell nuclear antigen (PCNA) was examined to evaluate the effects of Lin28a on the proliferation of mGSCs. Confirming our hypothesis, the expression level of PCNA in the OLin28a group was remarkably increased compared with that of the Con group (Fig. 5A,B). Moreover, the percentage of BrdU-positive cells in the OLin28a group was increased compared with that of the Con group (Fig. 5C).

**Figure 6:**
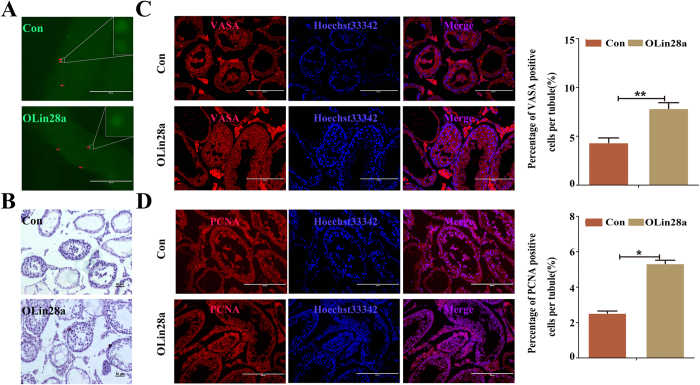
Transplantation experiment in mice injected with busulfan. (**A**) The transplanted cells were shown in the seminiferous tubules (red arrows). Up, transplanted GmGSCs-SB-pCDH cells. Down, transplanted OLin28a cells. (**B**) HE staining of mouse testes transplanted with different cells. The con group included transplanted GmGSCs-SB-pCDH cells, and the OLin28a group included transplanted the OLin28a cells. The OLin28a group had more cells in the seminiferous tubules, and the results indicated that the GmGSCs-SB-Lin28a cells proliferate faster. The two sections are 100x. Bar = 50 μm. (**C**) Immunofluorescent staining of VASA in the testes. Bar = 200 μm. (**D**) Immunofluorescent staining of PCNA in the testes. Bar = 200 μm.

To evaluate whether Lin28a promotes the self-renewal and proliferation of dairy goat mGSCs *in vivo,* the OLin28a group and Con group were transplanted into seminiferous tubules of busulfan-treated infertile mice. GFP-positive cells were observed after one month in seminiferous tubules of transplanted mouse testis (Fig. 6A), which also indicated that the transplantation system was successful. HE staining showed that there were more OLin28a cells in the seminiferous tubule (Fig. 6B). Immunofluorescence analysis showed that the testes transplanted with OLin28a cells have more VASA- and PCNA-positive germ cells compared with control (Fig. 6C,D). In conclusion, Lin28a promotes the self-renewal and proliferation of dairy goat mGSCs.

### Signaling mechanisms involved in Lin28a affects the proliferation of dairy goat SSCs

**Figure 7:**
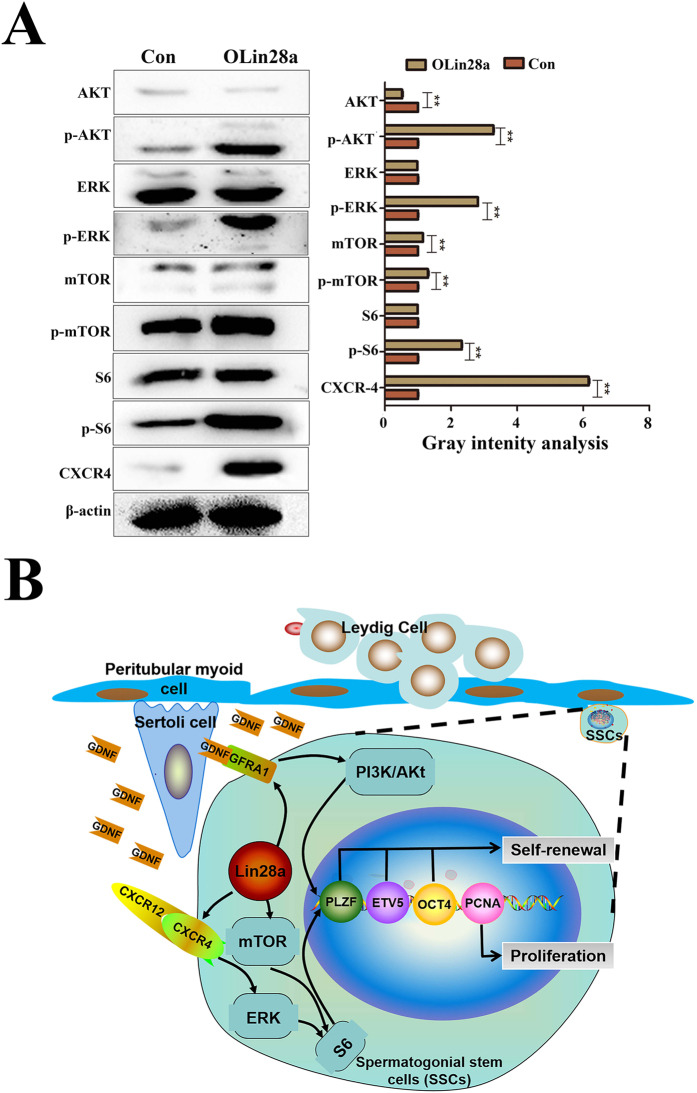
Signaling mechanisms involved in Lin28a stimulation. (**A**) Western blot analysis of the expression of AKT, p-AKT, ERK, p-ERK, mTOR, p-mTOR, S6, p-S6, and CXCR4. (**B**) A schematic of how Lin28a may affect the self-renewal of mGSCs. Lin28a activated Gfra1 and then activated the AKT signal pathway. Then, AKT activated the mTOR-S6 pathway to promote the expression of proliferation-related genes to maintain self-renewal or activate the expression of ETV5 to directly maintain self-renewal. By activating CXCR4, Lin28a activated the ERK pathway to promote the expression of proliferation-related genes to maintain self-renewal. **p < 0.01.

For the first time, we analyzed the signaling mechanisms involved in SSC proliferation and self-renewal regulated by Lin28a. Western blotting showed that phosphorylation of ERK1/2 was enhanced in the OLin28a group compared with the Con group (Fig. 7A). However, minimal differences in the expression level of ERK1/2 were noted between the two groups (Fig. 7A). Then, we analyzed the expression of CXCR4, a membrane protein receptor of CXCR12^27^. CXCR4 was highly expressed in Lin28a-overexpressing mGSCs compared with control (Fig. 7A). Therefore, we assumed that Lin28a increased the expression of the CXCR12-CXCR4 protein complex to efficiently enhance the phosphorylation of ERK1/2. ERK1/2 regulates downstream proliferation genes to promote the proliferation of dairy goat SSCs (Fig. 7B)^23^.

AKT and mTOR are key signals that regulate the balance between the self-renewal and differentiation of SSCs^20^. In our study, the expression of phosphorylated AKT was enhanced in a manner that was dependent on the increase of Lin28a (Fig. 7A). Therefore, we assumed that Lin28a enhances the expression of phosphorylated AKT. Phosphorylated AKT accelerates the transcription of ETV5, which was up-regulated to stimulate the self-renewal of Lin28a-overexpressing mGSCs (Fig. 7A). GFRA1 is the receptor of GDNF and plays an important role in SSCs^28^. Our results also showed increased GFRA1 protein in the membrane of the Olin28a group, which can efficiently absorb GDNF to maintain the self-renewal of the dairy goat SSCs (Fig. 4F). Thus, Lin28a up-regulated the expression of GFRA1 to improve the efficiency of AKT phosphorylation, which enhanced ETV5 expression and stimulated the self-renewal of mGSCs (Fig. 7B)^29^. Moreover, in this study, S6 phosphorylation was also enhanced (Fig. 7A), indicating that the mTOR (Fig. 7A) signal pathway was activated.

## Discussion

LIN28 is an essential RNA-binding protein that is highly expressed in ESCs but is significantly down-regulated in most differentiated adult tissues^30^. However, some tissues, such as skeletal muscle and cardiac tissue, persistently express LIN28^31^. Interestingly, we demonstrated that Lin28a is widely expressed in dairy goat tissues, with the high levels of expression noted in pubertal testis. The expression pattern of Lin28 was consistent with PLZF in dairy goats and was similar to that in mice and human^32^. Recently, it has been demonstrated that Lin28a is expressed in SSCs and may serve as a novel SSC marker in non-human primates (NHPs) and humans^33^. As expected, Lin28a may also be a marker in dairy goat SSCs as it is characteristically expressed only in SSCs of dairy goat testis at different ages.

Lin28a is highly conserved in different species. We used bioinformatics analysis software and a website to analyze our cloned dairy goat Lin28a. The results indicate that dairy goat Lin28a is located on chromosome 2 and encodes a 209-amino acid (aa) protein. The Lin28a protein contains two types of RNA-binding motifs: a N-terminal cold shock domain (CSD) and a C-terminal cys-cys-his-cys (CCHC) domain harboring a pair of retroviral-type CCHC zinc fingers^34^. Lin28b, the Lin28a paralog in humans, shares an overall protein identity of 77% with Lin28a and contains both conserved RNA binding motifs^35^. Lin28a cannot bind to mRNAs as a result of the deletion of the CSD domain or CCHC domain. These results suggest that both the CSD and CCHC domains are involved in mRNA-binding^36^.

Male germline stem cells (mGSCs), also known as SSCs, are the only type of adult stem cells that transmit genetic information to the next generation^36^. mGSCs have a self-renewal capacity to maintain the balance between the number of mGSCs and differentiation to produce sperm. The self-renewal potential is impaired in mouse ESCs if we knockdown the expression of Lin28a^10^. Lin28a is expressed in spermatogonia of involuted rodent and primate testes^37^,^38^, which are known to express many pluripotent stem cell markers^39^,^40^. Furthermore, Lin28a is essential for germ cell specification in mice. Hence, we hypothesize that the highly conserved RNA-binding protein is also involved in maintaining the identity of adult SSCs and promotes the self-renewal and proliferation of dairy goat SSCs. To test the hypothesis, we overexpressed Lin28a in mGSCs-I-SB cells^41^. The expression of the pluripotency factors SOX2 and OCT4 is up-regulated by overexpression of Lin28a. These findings indicated that the pluripotency of mGSCs-I-SB cells is enhanced with increased Lin28a expression. Additionally, the markers of SSC self-renewal, GFRA1, PLZF and ETV5, are also up-regulated. Therefore, we suggest that Lin28a overexpression in mGSC-I-SB cells can accelerate mGSC self-renewal. Cell cycle, PCNA expression and BrdU incorporation analyses suggest that overexpressed Lin28 promotes the proliferation of dairy goat mGSCs.

Many studies have provided compelling evidence that Lin28a directly binds to mRNA and acts as a post-transcriptional regulator^6^,^7^,^9^. Studies in skeletal myoblasts confirmed the interaction between Lin28 with IGF2^6^. Furthermore, data revealed that Lin28a binds the coding region of histone H2a mRNA in mouse ESCs and the coding region of OCT4 mRNA^7^,^9^. In this study, we hypothesize that Lin28a binds to Plzf via the GGAGA motif enriched within exons and the 3-UTR of PLZF. However, there is no direct interaction between Lin28a and PLZF as analyzed by the luciferase reporter assay (unpublished data). Thus, we hypothesize that Lin28a regulates PLZF in an indirect manner. Recent studies showed that Lin28a directly binds to a consensus DNA sequence *in vitro* and in mouse ESCs *in vivo*^42^. Further, the mechanism by which Lin28a affects dairy goat mGSCs must be elucidated by ChIP-seq and RNA-seq analysis as well as conventional methods.

The mTOR and ERK signaling pathways play a role in the proliferation and stimulation of meiotic initiation of SSCs^42^,^43^. Phospho-S6 Ribosomal Protein (Ser235/236) was up-regulated by Lin28a in dairy goat mGSCs. Growth factors and mitogens effectively promote sustained cell growth and proliferation by up-regulating mRNA translation^44^,^45^,^46^. Growth factors and mitogens induce the activation of p70 S6 kinase and the subsequent phosphorylation of the S6 ribosomal protein. Phosphorylation of the S6 ribosomal protein correlates with an increase in translation of mRNAs transcripts that contain an oligopyrimidine tract in their 5′ untranslated regions^44^. These particular mRNA transcripts (5′TOP) encode proteins that are involved in cell cycle progression as well as ribosomal proteins and elongation factors that are necessary for translation^44^,^47^. S6 ribosomal protein phosphorylation sites include several residues (Ser235, Ser236, Ser240, and Ser244) located within a small carboxy-terminal region^47^,^48^. To explore how Lin28 promotes the proliferation of dairy goat mGSCs, we tested the mTOR and ERK signaling pathways in overexpressed Lin28 mGSCs. The results suggest that Lin28 activated pERK1/2, p-AKT and p-mTOR phospho-S6 ribosomal proteins (Ser235/236).

Taken together, this study demonstrated that Lin28a is widely expressed in dairy goat tissues and is located in the SSC cytoplasm. Overexpression of Lin28a accelerates mGSC proliferation through up-regulation of PCNA, OCT4, SOX2, GFRA1 and PLZF and activation of the pERK1/2, p-AKT and mTORC1 pathways. To the best of our knowledge, this is the first study that reports the function of Lin28-mediated regulation on the self-renewal and proliferation of dairy goat SSCs through regulation of the mTORC and PI3K/AKT pathways.

## Materials and Methods

### Cells and collection of Capra hircus tissues

Dairy goat male germline stem cells (GmGSCs) were stored in Shaanxi Centre of Stem Cells Engineering and Technology, Northwest A&F University. These cells were primary cells isolated from dairy goat testes that were transduced with SV40 large T antigen and Bmi1 to establish immortalized male goat germline stem cells (GmGSC-I-SB)^41^.

Guanzhong dairy goat testes at different ages (3, 6, 9, 10, 12 and 24 month) were supplied by Yaoan slaughterhouse in the Yangling Hitech area.

### Ethics statement

All animal experiments were performed in strict accordance with the the Guide for the Care and Use of Laboratory Animals (Ministry of Science and Technology of the People’s Republic of China, Policy No. 2006 398) and were approved by the Animal Care and Use Center of the Northwest A&F University.

### Semi-quantitative RT-PCR analysis

Total RNA was extracted from different tissues of adult dairy goats, and testicular tissues at different ages (3, 6, 9, 10, 12 and 24 month) were detected by semi-quantitative RT-PCR analysis according to the manufacturer’s instructions (Tiangen, China). The RNA concentration and purity were evaluated by a NanoDrop 2000 spectrophotometer (Thermo Scientific, Pittsburgh, PA, USA). cDNA was synthesized using a commercially available kit (Thermo).

For each sample, cDNA was synthesized using 1 μg of RNA and a commercially available kit (Thermo Scientific First Strand cDNA Synthesis Kit). The PCR steps included denaturation at 95 °C for 5 min; 30 cycles at 95 °C for 30s, 58 °C for 30s, and 72 °C for 30s, and a final extension at 72 °C for an additional 10 min. The RT-PCR primers used are described in Table S2. The PCR products were analyzed in 1% agarose (Invitrogen, CA) gel electrophoresis, stained with ethidium bromide (Invitrogen), and visualized under UV illumination^49^. Glyceraldehyde-3-phosphate dehydrogenase (GAPDH) was used as the internal control.

### Real-time quantitative PCR analysis (RT-QPCR)

Total RNA of dairy goat testicular tissues at different ages and other indicated tissues were extracted and measured concentration and purity by NanoDrop 2000 spectrophotometer (Thermo Scientific, Pittsburgh, PA, USA). Briefly, 1 μg of RNA was used to synthesize cDNA by using Reverse Transcription Kit (Thermo Scientific First Strand cDNA Synthesis Kit).

Lin28a and other relevant gene expression levels in the GmGSCs-I-SB-GFP cells and GmGSCs-I-SB-Lin28a cells were detected by the CFX96TM Real-Time System (C1000TM; Bio Rad Thermal Cycler, Foster City, CA, USA). Briefly, 0.5 μl of cDNA was used for each 15-μl PCR reaction with 7.5 μl of SYBR Green Mastermix (Bioer Co. Ltd., Hangzhou, China), 6.3 μl of ddH2O, 0.3 μl of sense primer, 0.3 μl of antisense primer, and 0.1 μl of TaqDNA polymerase. Each sample was assayed in triplicate. The PCR program consisted of initial denaturation (5 min at 95 °C), followed by 40 cycles denaturation (20 s at 95 °C) and annealing (30 s at 58 °C) and elongation (10s at 72 °C) steps. The expression levels of each sample were normalized to GAPDH. The 2[–ΔΔC(T)] method was used to analyze the relative quantification^50^.

RT-QPCR primers used are listed in Supplementary Table S2.

### Immunofluorescence staining

Testicular tissues obtained were fixed in 4% paraformaldehyde (PFA) and embedded in paraffin. Embedded blocks were sectioned at 2 μm and were immersed in xylene I and II for 8 min, separately. Next, the samples were successively immersed in 100%, 95%, and 75% alcohol for 5 min each and finally rinsed in deionized water for 5 min. Antigen retrieval was achieved in boiling sodium citrate buffer for 10 to 15 min, and then, the samples were washed three times in 1 × PBS for 5 min each after they cooled to room temperature (RT). Then, the samples were blocked in blocking solution (1 × PBS + 1% BSA) for 30 min and incubated with primary antibody against Lin28a (1:50, Santa Cruz) overnight at 4 °C. Sections were then washed in 1 × PBS three times and incubated with a goat anti-rabbit IgG antibody conjugated to Alexa Fluor 488 (Invitrogen) for 1 h at RT. Coverslips were mounted onto slides with Anti-fading Buffer mounting medium (Bioworld) containing 1 μg/ml Hoechst33342 (Sigma)^49^,^51^.

### Alignment and evolutionary relationship among the Lin28a CDS

The dairy goat Lin28a CDS was obtained and sequenced by Sangon China. Multiple sequence alignment identified Lin28a among different species using DNAman software, and the phylogenetic tree was depicted with MEGA4.1^51^.

### Alignment of the Lin28a proteins

The amino acid sequences in different species were also analyzed by DNAMAN software. The domains contained in Lin28a protein were predicted by SWISS-MODEL Workspace website and RasMol software^52^.

### Construction of recombination plasmid

The primer sequences for dairy goat Lin28a CDS clone were designed according to the published *Bos taurus* Lin28a mRNA sequence (XM_005895879.1) as follows:

Forward-5′-GGAATTCAATGGGCTCTGTGTCAAACC-3′,

Reverse-5′-GGGATCCTCTGTGGCTTCAATTCTGG-3′.

Lin28a was amplified from the dairy goat testicular cDNA by reverse transcription polymerase chain reaction (RT-PCR)^53^. Then, the specific fragments were cloned into the pMD18-T cloning vector (TaKaRa, Dalian, China). After selection by ampicillin, the positive clones were verified by PCR, double restriction enzyme digestion and sequencing. Then, the fragments were inserted into the pCDH-CMV-MSC-EF1 eukaryotic expression vector to obtain the pLin28a-CDH-CMV-EF1 recombination plasmid. Verification was performed by PCR and double restriction enzyme digestion by EcoRI and BamHI.

### Lentivirus preparation and cell transduction

The lentivirus system was modified as reported by Anokye-Danso *et al*.^54^. GmGSCs-I-SB cells were seeded in a 6-well plate at a density of 1 × 10^5^ cells twelve h before transduction. Cells expressing the GFP protein were considered to be successfully transduced. Positive cells were purified by 500 ng/ml puromycin dihydrochloride, and the efficiency of purification was evaluated by the GFP expression using flow cytometry (FCM).

### Flow cytometry analysis

The cells were seeded in 48-well plates overnight with a density of 2 × 10^5^ cells. The GFP-positive rate of the cells was detected by a Beckman Coulter flow cytometer (Beckman Coulter, Brea, CA, USA) using GmGSCs-I-SB as the blank control^23^.

### Cell immunofluorescence staining

GmGSCs-I-SB-GFP cells and GmGSCs-I-SB-Lin28a cells were fixed in 4% paraformaldehyde (PFA) for 15 min and treated with 0.1% Triton X-100 for 10 min at room temperature^55^. Then, the samples were blocked with 1% BSA for 30 min and incubated in primary antibody at 4 °C overnight. The following primary antibodies were used: LIN28A antibody (1:200, Santa Cruz), SOX2 antibody (1:100, Bioss), OCT4 antibody (1:100, Bioss), PLZF antibody (1:100, Bioss), and GFRA1 antibody (1:100, Bioss). Goat anti-rabbit IgG antibodies conjugated to Alexa Fluor 488 (1:500; Invitrogen) were incubated for 1 h at room temperature in the dark. Cell nuclei were stained by Hoechst 33342 (Sigma) at room temperature (RT) for 5 min. The samples were washed by PBS three for 5 min. Images were analyzed by a EVOS^®^ Imaging System.

### Western blotting analysis

Protein was extracted from GmGSCs-I-SB cells transduced with Lin28a or control by using sodium dodecyl sulfate polyacrylamide gel electrophoresis (SDS-PAGE) sample loading buffer (Beyotime). Then, total cell protein was resolved by SDS-PAGE followed by transfer to a polyvinylidene difluoride membrane. The membranes were incubated with primary antibodies, including the LIN28A antibody (1:500, Santa Cruz), SOX2 antibody (1:200, Bioss), PCNA antibody (1:1000, Biolegend), OCT4 antibody (1:200, Bioss), ETV5 antibody (1:1000, abcam), GFRA1 antibody (1:400, Santa Cruz), ERK antibody (1:1000, Cell Signaling Technology), p-ERK antibody (1:500, Cell Signaling Technology), AKT antibody (1:1000, BOSTER, Wuhan, China), p-AKT antibody (1:500, Sangon), S6 antibody (1:1000, Cell Signaling Technology), mTOR antibody (1:1000, Cell Signaling Technology), p-mTOR antibody (Ser2448, 1:1000, Cell Signaling Technology), pS6 (Ser235/236, 1:1000, Cell Signaling Technology), CXCR4 antibody (1:400, Bioss), and anti-β-actin antibody (1:1,000, Cell Signaling Technology). Horseradish peroxidase-conjugated anti-rabbit or mouse IgG antibodies were used as secondary antibodies (1:1000, BOSTER). Detection was performed using the Chemistar Hiht-sig ECL Western Blotting Substrate (Tanon). The results were analyzed by Tanon-410 automatically image system (Shanghai Tianneng Corporation, China).

### BrdU incorporation assay

Cells were incubated with 30 μg/ml BrdU (Sigma, St Louis, MO, USA) for 6 h and then subjected to BrdU immunostaining. The cells were fixed in formaldehyde and acetone (1:1) for 15 min and then treated with 0.1% Triton X-100 for 10 min at room temperature. Then, the samples were incubated in BrdU (1:100; Santa Cruz) primary antibody at 4 °C overnight. The next steps were the same as those reported for cell immunofluorescence staining^23^.

### Transplantation and immunohistochemistry

Ten 7-week-old male ICR mice were purchased from the animal center of the Fourth Military Medical University in Xi’an, China. The mice were injected in the abdomen with busulfan at 30 mg/kg (>25 g) to gain infertile mice. Three weeks after busulfan treatment^56^, 7 of the infertile mice were separately microinjected in the seminiferous tubules with the GmGSCs-I-SB cells (Con) and Lin28a-overexpressing (OLin28a) cells (1 × 10^4^/testis). The cells were suspended with culture medium. Three of the infertile mice were separately microinjected with moderate culture medium in the seminiferous tubules. One month later, the mice were sacrificed by cervical dislocation. The bilateral testes were harvested and fixed with 4% formaldehyde overnight. The 2-μm sections were processed for immunostaining. The following primary antibodies were used: VASA antibody (1:500, abcam) and PCNA antibody (1:100, BOSTER, Wuhan).

### Statistical analysis

Student’s t-test was used to analyze the statistical differences between groups. All data were presented as the mean ± SD from three different experiments and were analyzed using Graphpad Prism software (La Jolla, CA, USA) *P < 0.05. **P < 0.01.

## Additional Information

**How to cite this article:** Ma, F. *et al*. Lin28a promotes the self-renewal and proliferation of dairy goat spermatogonial stem cells (SSCs) through regulation of mTOR and PI3K/AKT. *Sci. Rep.* **6**, 38805; doi: 10.1038/srep38805 (2016).

**Publisher's note:** Springer Nature remains neutral with regard to jurisdictional claims in published maps and institutional affiliations.

## Supplementary Material

Supplementary Information
