# Supplementary material for: Lin28a promotes self-renewal and proliferation of dairy goat spermatogonial stem cells (SSCs) through regulation of mTOR and PI3K/AKT
Source: Sci Rep. 2016 Dec 12;6:38805. doi: 10.1038/srep38805 (PMC5150521; doi:10.1038/srep38805)
Supplement: Supplementary Information [file srep38805-s1.pdf]

Supplementary Information

Lin28a promotes self-renewal and proliferation of dairy goat spermatogonial stem cells (SSCs) through regulation of mTOR and PI3K/AKT

Fanglin Ma1, Zhe Zhou1, Na Li1, Liming Zheng1, Chongyang Wu1, Bowen Niu1 , Furong Tang1, Xin He1, Guangpeng Li2*, Jinlian Hua1*

1. College of Veterinary Medicine, Shaanxi Centre of Stem Cells Engineering & Technology, Northwest A&F University, Yangling, Shaanxi, 712100 China

2. Key Laboratory for Mammalian Reproductive Biology and Biotechnology, Ministry of Education, Inner Mongolia University, Hohhot, 010021, China

Atgggctctgtgtcaaaccagcagttcgcaggtggctgcgctaaggcgccggaggaggcgccggaggaggcgccgg aggacgcggcccgcgcggcggaggagccgcagctgctgcacggtgccggcatctgtaagtggttcaacgtgtgcatgg ggttcggcttcctgtccatgaccgcccgcgcaggggtcgcgctcgaccccccggtggatgtctttgtgcaccagagtaagc tgcacatggagggcttccggagcctgaaggagggggaggctgtggagttcacctttaagaagtccgccaagggcctgga atctatccgagtcaccggccctggtggggtgttctgtattgggagtgaaaggcggcccaaagggaagaatgtgcagaaac gcagatcaaagggagacaggtgctacaactgtggaggtctagaccatcatgccaaggaatgcaaactgccaccgcagcc caagaagtgccatttctgccagagcatcagccatatggtagcctcgtgcccactgaaggcccagcaagctcccagctccc agggaaagccagcctactttcgggaggaggaagaagagatccatagctctgccatgctcccagagacccagaattga

Figure S1 **The sequence of *Capra hircus* Lin28a**

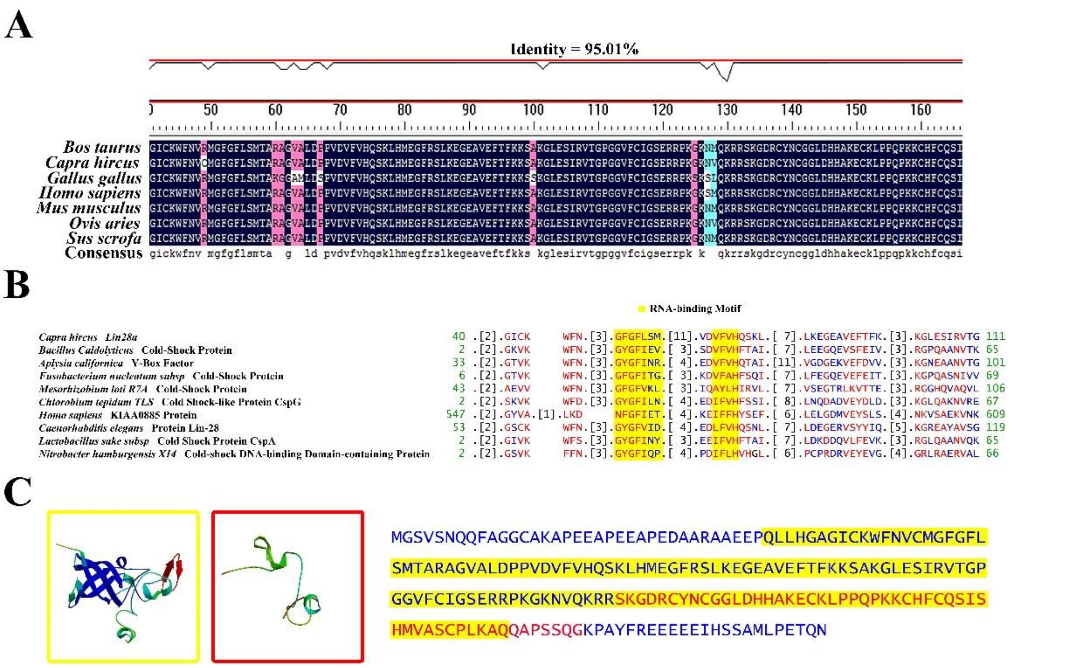

Figure S2 **Bioinformatic analysis of Lin28a.**

A, Similarity comparison of Lin28a amino acid sequence showed that Lin28a is conserved; B, Multiple sequence alignment of *Capra hircus* Lin28a and the protein sequences used to curate the domain model, where yellow marks overlaping the aligned sequences show the location of the conserved feature residues of RNA-binding Motif; C. The predicted model in yellow box of *Capra hircus* Lin28a from 34 to 179 amino acids, which contains an S1-like cold-shock domain (CSD), are thought to bind mRNA and regulate ribosomal translation, mRNA degradation, and the rate of transcription termination. Red box model is the two the Zinc-finger domains of *Capra hircus* Lin28a from 134 to 186 amino acids, recognised to bind downstream target mRNA.

Table S1 The length comparison of Lin28a of CDS and amino aid

Species	Length of CDS	Length of amino acid	Homology (%)
Capra hircus	630	209	100%
Bos taurus	618	205	95.61%
Gallus gallus	609	202	75.24%
Homo sapiens	630	209	90.84%
Mus musculus	630	209	89.52%
Ovis aries	630	209	99.52%
Sus scrofa	618	205	92.38%

Table S2 The primer sequences

Gene name	Primer	Tm/℃	Size/bp
Lin28a	Forward: GGTTCGGCTTCCTGTCCAT	58	126
	Reverse: CAACTGCCTCACCCTCCTT		
Plzf	Forward: CACCGCAACAGCCAGCACTAT	58	127
	Reverse: CAGCGTACAGCAGGTCATCCAG		
Gfra1	Forward: GGACAGGCAGCAGGAAATA	58	200
	Reverse: GTCTCCTGTCCCAGTCAAA		
Sox2	Forward: GGCGGCAACCAGAAGAACAG	58	109
	Reverse: GCATCTTGGGGTTCTCCTGG		
Oct4	Forward: AAGCAGTGACTACTCCCAACG	58	155
	Reverse: GGGAATGGGACCGAAGAGTA		
Pcna	Forward:AGTGGAGAACTTGGAAATGGAA	58	154
	Reverse: GAGACAGTGGAGTGGCTTTTGT		
Gapcdh	Forward: CCACGCCATCACTGCCACCC	58	116
	Reverse: CAGCCTTGGCAGCGCCAGTA		
